# The Effectiveness and Common Features of Reablement Services on Clients’ Daily Functioning: A Systematic Review

**DOI:** 10.1093/geroni/igab046.1612

**Published:** 2021-12-17

**Authors:** Lise Buma, Stan Vluggen, Sandra Zwakhalen, Gertrudis Kempen, Silke Metzelthin

**Affiliations:** Maastricht University, Maastricht, Limburg, Netherlands

## Abstract

This systematic review, guided by the ReAble-definition, (1) provides an overview of reablement interventions and their effect on Activities of Daily Living (ADL), and (2) identifies common features of effective interventions. A systematic search was conducted from 2002 to 2020, identifying nineteen studies from eight countries with a total of 6,534 participants. Ten studies (with moderate to high quality) revealed improvements in ADL functioning. Three common intervention features were identified within effective interventions: use of multidisciplinary teams; a protocoled or standardized assessment; and using multiple components such as ADL-training, education and exercise programs. This review emphasizes that future studies should provide a more consistent and detailed reporting on the intervention and its components. Furthermore, a uniform approach regarding components, follow-up times and outcome measures can contribute to the comparison of reablement interventions and better determine their effectiveness, independent of the healthcare system or country in which it is used.

